# Perceived Impact of an Online Community Care Platform for Dutch Older Adults on Local Participation, Informal Caregiving, and Feelings of Connectedness: Pretest-Posttest Observational Study

**DOI:** 10.2196/20304

**Published:** 2020-12-01

**Authors:** Sarah Willard, Erik van Rossum, Marieke Spreeuwenberg, Luc de Witte

**Affiliations:** 1 Centre of Innovative Care and Technology (EIZT) Zuyd University of Applied Sciences Heerlen Netherlands; 2 Care and Public Health Research Institute Faculty of Health, Medicine and Life Sciences Maastricht University Maastricht Netherlands; 3 Centre for Assistive Technology and Connected Healthcare University of Sheffield Sheffield United Kingdom

**Keywords:** older adults, online community, online platform, social network, local participation, informal caregiving

## Abstract

**Background:**

In a changing ageing society wherein older adults are increasingly expected to take care of themselves instead of relying on health care services, online community care platforms can help older adults to meet these expectations. A considerable number of these online community care platforms have been introduced in several European countries based on their potential. However, their actual impact is unclear.

**Objective:**

The aim of this study was to investigate the self-reported use, expectations, and perceived impact of a Dutch online community care platform called Grubbenvorst-Online among Dutch older adults. The following 2 questions were studied: (1) What is the self-reported use of Grubbenvorst-Online among older adults? (2) What are their expectations and perceived impact of Grubbenvorst-Online regarding local participation, their social network, mutual informal caregiving, and feelings of connectedness?

**Methods:**

An observational pretest-posttest study was conducted. Participants were recruited via a web-based message on the Grubbenvorst-Online platform and data were collected via postal questionnaires among older users at the start of the study and 4 months later. Data regarding the expectations and the perceived impact of Grubbenvorst-Online were compared and tested.

**Results:**

Forty-seven Grubbenvorst-Online users with an average age of 74 years participated in this study. They were healthy, predominantly “internet-skilled,” and they found the internet important for maintaining social contacts. In general, the use of the online community care platform decreased during the 4-month follow-up period. The perceived impact of Grubbenvorst-Online was significantly lower than that expected regarding information provision (*P*=.003), seeking help from fellow villagers (*P*<.001), giving help to fellow villagers (*P*<.001), and consulting care or welfare services (*P*<.001).

**Conclusions:**

The findings of this study indicate that online community care platforms perhaps do not provide enough “added value” in their current form. We suggest a new direction in which online community care platforms primarily support existing offline initiatives aimed at stimulating local participation, informal caregiving, and feelings of connectedness.

## Introduction

In a changing ageing society wherein older adults are increasingly expected to take care of themselves instead of relying on health care services [1,2], online community care platforms can help older adults to meet these expectations. A considerable number of web-based platforms for older adults has been introduced in several European countries, for example, Germany [3], the United Kingdom [4], Belgium, and the Netherlands [5-8]. All these platforms target older adults and their care networks to facilitate and support aging-in-place.

In our scoping review, a typology of online care platforms for community-dwelling older adults was developed. This review was performed because little research had been conducted on the availability of web-based platforms for older adults and their characteristics, functionalities, and usability in order to guide older adults in choosing a suitable platform. The review resulted in an overview of 21 care platforms, which can be classified into the following 3 types: (1) *Online Community Care Platforms*, which attempt to enhance social cohesion by interlinking community-dwelling older adults with neighboring informal caregivers and by promoting local activities at the neighborhood level; (2) *Online Care Network Platforms*, which provide older adults and professional and informal caregivers tools to coordinate, plan, and communicate about (health) care; and (3) *System Integrator Platforms*, which interconnect a variety of functionalities. The latter platform type has the capability of integrating existing services and apps into its own software, that is, it operates as an “empty” information communication technology framework, which can be filled with any content [9]. This study focuses on a *System Integrator Platform* that was deployed as an online community care platform. In other words, all functionalities in this information communication technology framework were aimed at enhancing social cohesion or at promoting local activities at the neighborhood level. Thus, we choose to use the term “online community care platform” when referring to the platform in this study.

Online community care platforms offer older adults various apps aimed at supporting their independent living (eg, products and services) and civic and social participation (eg, contacts, messages, a matching tool for informal care). Studies have shown that more common web-based communities for older adults, which are known under different names such as “social networking sites” (eg, Facebook, Twitter, LinkedIn), “online social networks,” or “online social communities” can potentially have a positive impact on civic participation and help to develop and maintain social relationships and social support [10-13].

It seems that online community care platforms are implemented in several European countries based on the assumption that they can support older adults to age-in-place, to participate locally, and that they can help to develop and maintain social relationships and to arrange social support. As governments call for increased autonomy at local levels [1], these platforms seem perfectly suited tools for older adults to actually remain or even become more autonomous. However, their *actual* (perceived) impact is unclear [9]. The majority of previous research has focused on the usability and acceptability of these web-based communities or platforms and thus on the preimplementation phase. Hardly any studies report the factors that contribute to the continued use or to the desertion of web-based communities once they have been implemented [14]. It is important for both users and policy makers to discover to what extent these platforms actually help older adults to participate locally and socially.

**Figure 1:**
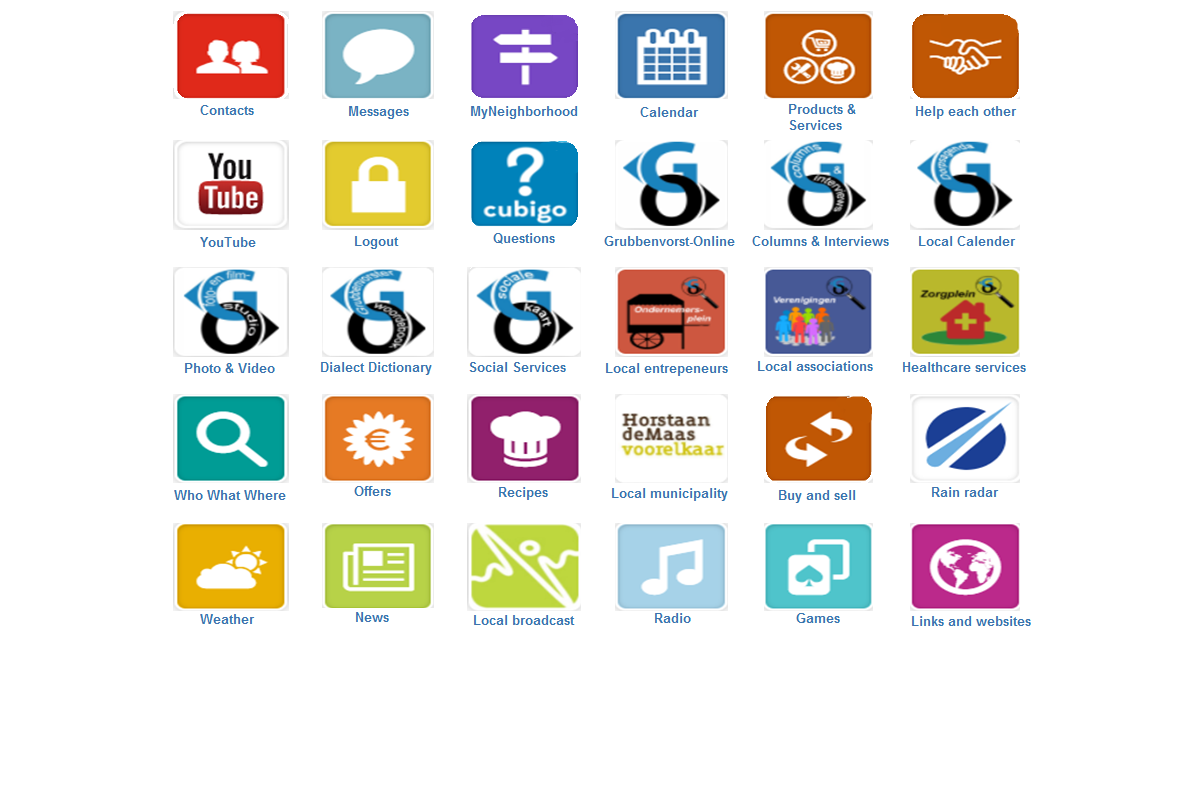
Screenshot of the online community care platform Grubbenvorst-Online.

This study therefore primarily focusses on the perceived impact in the postimplementation phase of an online community care platform that was implemented in 2015, called as “Grubbenvorst-Online” (abbreviated as GO, Grubbenvorst refers to the town in which the platform was implemented). Grubbenvorst-Online was the initiative of an active group of local older citizens (“the initiators”) who, through the platform, aimed to help both older adults and vulnerable inhabitants of Grubbenvorst to socially participate locally. Grubbenvorst- Online was founded by the initiators in close collaboration with local entrepreneurs, associations, and social and health care organizations. Arestoco, a Belgian enterprise, provided the information communication technology framework for the Grubbenvorst-Online platform. Their platform entitled “Cubigo” was selected, as its software could be modified based on the wishes and needs of potential users [15]. The content of Grubbenvorst-Online (Figure 1) was determined and kept up-to-date by the initiators.

**Figure 2:**
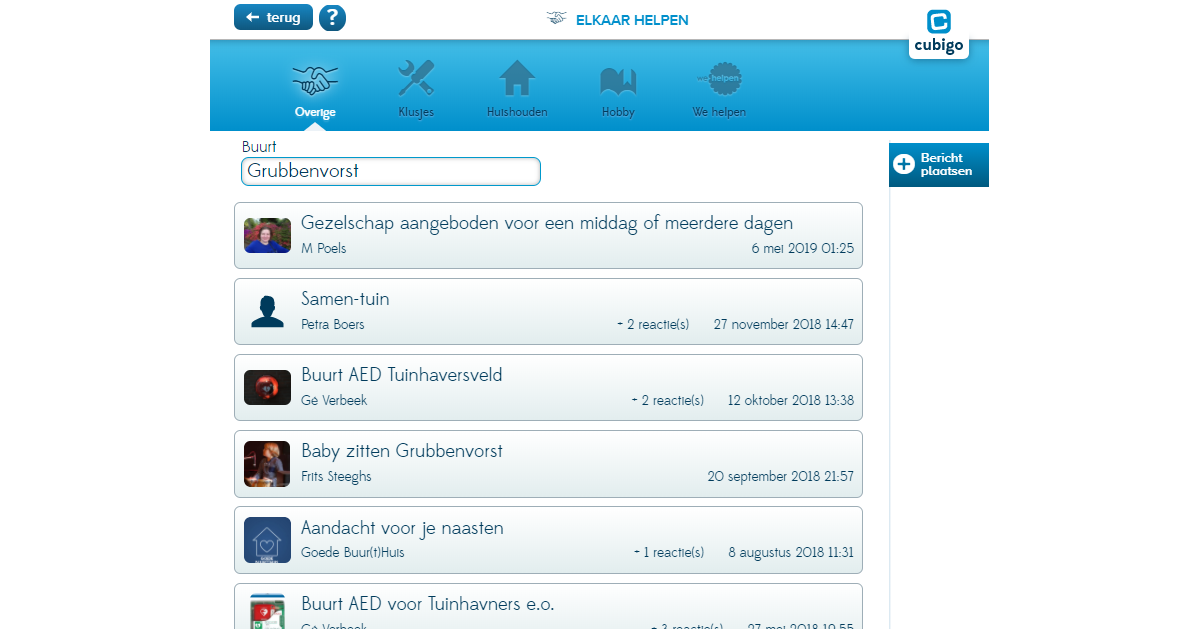
Screenshot of the functionality "Help each other".

**Figure 3:**
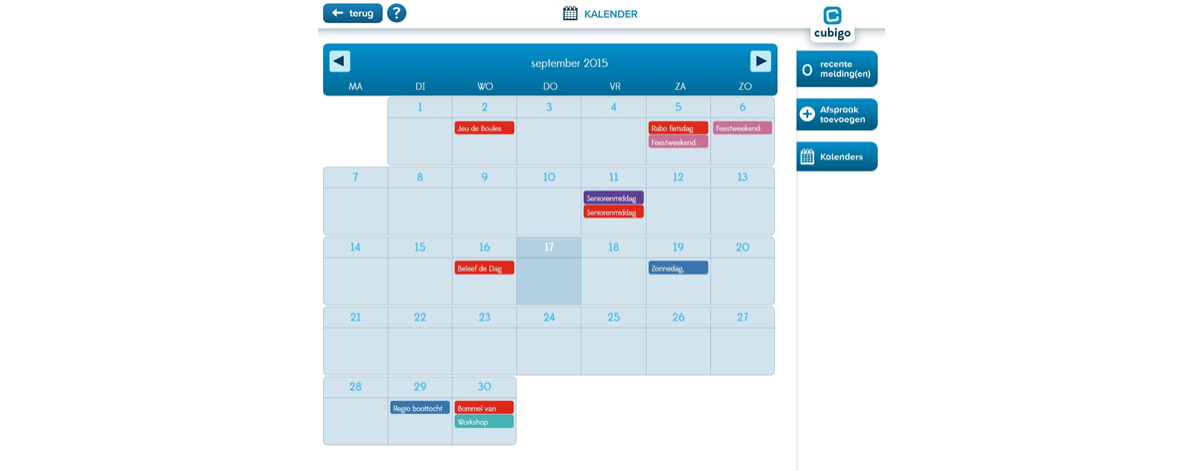
Screenshot of the functionality "Calendar".

A few examples of the platform’s apps are (1) a matching tool for informal care, called as “Help each other,” in which users can exchange informal help (Figure 2), (2) a local calendar where information can be found on local events and activities (Figure 3), and (c) social services in which users can find information about available care services and organizations.

The following 2 questions were studied.

1. What is the self-reported use of Grubbenvorst-Online among older adults?
2. What are the expectations and perceived impact of Grubbenvorst-Online among older adults regarding local participation, their social network, mutual informal caregiving, and feelings of connectedness?

We wanted to determine what users expected from Grubbenvorst-Online when they started using it, what did they think the platform was intended for and had to offer in general, and then to investigate to what extent the platform met these expectations.

## Methods

### Design

This study had an observational pretest-posttest design. Data on the use, expectations, and perceived impact regarding the online community care platform Grubbenvorst-Online was collected via 2 postal questionnaires completed by older users. Our aim was to perform an exploratory study among approximately 50 participants.

### Setting

Grubbenvorst is a sparsely populated village in Limburg, which is the southernmost province of the Netherlands. Approximately 1200 of the 4800 inhabitants (25.0%) are 65 years or older [16]. Grubbenvorst-Online was made available for all inhabitants of Grubbenvorst in August 2015. Several activities were undertaken to communicate and promote the existence and availability of the platform, such as (1) public launch of Grubbenvorst-Online in the central square in Grubbenvorst in the presence of the alderman; (2) publication of advertisements in “Announcements” (a local newspaper); (3) distribution of flyers, brochures, and other public relations material; (4) placement of a large billboard at the main entrance of the village; (5) various informative presentations for organizations such as associations for older adults, care organizations, and an elementary school; and (6) taking part in a TV interview at the local broadcaster (TV Reindonk). At the start of the study in early 2018, Grubbenvorst-Online had approximately 725 users.

### Participants

Participants were recruited among the users in January 2018 via a web-based message on the Grubbenvorst-Online platform. This message described the purpose of the study, that is, to gather information regarding the way in which older adults used the platform and how they perceived its impact. Older adults were asked, if interested, to register within 1 month by leaving their contact details with the initiators. Applicants were suitable for inclusion if they were 65 years or older and if they were a user of the online community care platform. This means that every potential participant was an existing user, had already registered on the online community care platform, and was therefore more or less familiar with the functionalities and operation of the platform. Furthermore, the length of time that the participants used the platform was *not* an inclusion criterion; hence, both “old” and “new” users were suitable for inclusion. Recruitment stopped by the time 51 participants had registered.

### Statement of Ethical Approval

The ethical principles that are outlined in the Dutch “Medical Research Involving Human Subjects Act” were followed throughout the entire study. The ethical approval for this study was given by METC-Z (16-N-213).

### Data Collection

Two postal questionnaires were used to collect reports of older adults regarding the use, expectations, and perceived impact of the Grubbenvorst-Online platform. Potential participants received an invitation letter, informed consent form, and the first (baseline) postal questionnaire (T0) in March 2018. Four months later, in July 2018, they received the second postal questionnaire (T1). Table 1 illustrates the themes and topics of the questionnaire and their operationalization. The baseline questionnaire (T0) was primarily designed to collect information on the *expectations* of older adults regarding the online community care platform: did participants expect the platform to have positive effects on local participation, informal caregiving, their social network, and feelings of connectedness? The follow-up questionnaire (T1) aimed to discover to what extent the expectations as formulated in T0 were met (ie, perceived impact). As there were no validated scales available to measure the expectations and perceived impact of online community care platforms, the scale was developed by us.

General characteristics of the participants were collected as well as details of their use of the internet and technology and use of informal care and their community involvement and related needs. Regarding the aforementioned characteristics, validated scales or parts of existing questionnaires were used. For topics 2-4 (Table 1), parts of the validated questionnaire “Senior Monitor Heerlen” [17] were used, that is, “Civic participation and social network” and “Living and environment.” Additionally, for topic 4, the validated scale “Involvement with neighbors” [18] was adopted. This scale incorporates 3 domains of social cohesion: (1) collaboration to stimulate local well-being; (2) solidarity: the extent to which neighbors help each other, and (3) feelings of connectedness: the extent to which neighbors feel connected to each other.

Concerning the topic of informal care, participants could answer yes or no to whether they gave help, whether they were willing to give help, whether they already received help, and finally, whether they would accept help from someone living in their close vicinity. If participants indicated that they helped someone, they did not have to answer the question whether they were prepared to offer help. The topic was introduced as follows: “the following questions are about your ability to help others or to receive help yourself. This “help” can, for example, consist of doing chores around the house or doing groceries together with family, friends, neighbors, or acquaintances. Thus, the questions were not related to physical care tasks.

**Table 1:** Questionnaire themes and their operationalization.

Theme	T0/T1^a^	Items (n)	Topics	Adopted propositions and questions
Participant characteristics	T0	27	(1) General characteristics (eg, gender, date of birth), (2) Use of internet and technology, (3) Informal care, (4) Community involvement and related needs	Examples of questions for topics 2-4: (2) How important is the internet for you to keep in touch with other people? (nominal 5-point scale from “very important” to “unimportant”), (3) Would you accept help from a neighbor? (Yes/No), (4) Do you feel involved with the people living in your immediate vicinity? (nominal 5-point scale: “With almost none,” “With most not,” “With some,” “With most,” “With almost everyone”).
Self-reported use of Grubbenvorst-Online	T0-T1	14-17^b^	Use of platform in general and of specific functionalities^c^	Examples of questions (nominal 4-point scale: “Never,” “Occasionally,” “Regularly,” and “Daily”): (1) How often do you currently use the Grubbenvorst-Online platform? (T0 + T1); (2) How often do you currently use the “Grubbenvorst Village Calendar”? (T0+T1); (3) Have you used Grubbenvorst-Online less, more often, or to the same extent compared to 4 months ago? (T1)
Expectations of Grubbenvorst-Online	T0	7	Expectations regarding the platform’s added value regarding local participation, informal caregiving, social networking, and feelings of connectedness	Examples of propositions (3-point scale: “[Strongly] Agree,” “Neither agree nor disagree,” “[Strongly] Disagree”): (1) I expect I will partake more in village activities because of Grubbenvorst-Online, (2) I expect that I will use Grubbenvorst-Online to ask my fellow villagers for help (eg, with a job around the house or grocery shopping)
Perceived impact of Grubbenvorst-Online	T1	7	Perceived impact of the platform regarding local participation, informal caregiving, social network, and feelings of connectedness	Examples of propositions (3-point scale: “[Strongly] Agree,” “Neither agree nor disagree,” “[Strongly] Disagree”): (1) Because of Grubbenvorst-Online, I partake more often in village activities. (2) I asked for the help of a fellow villagers via Grubbenvorst-Online (eg, for a job in the house or grocery shopping)

^a^T0: baseline questionnaire, T1: questionnaire after 4 months of the study.

^b^Apart from a few additional questions in T1 (such as example 3), the questions were identical in T0 and T1.

^c^Not all functionalities of Grubbenvorst-Online (see Figure 1) were assessed. Only functionalities identified by the initiators as most important were included in the questionnaire.

### Data Analysis

The baseline characteristics of the participants who filled out both questionnaires (T0 and T1) were expressed in mean (SD) or n (% of the participants). The comparison of the expectations and perceived impact of Grubbenvorst-Online was tested with the McNemar test. All *P* values were two-sided and were considered to be statistically significant if less than .05. SPSS 25 (IBM SPSS Statistics for Windows, IBM Corp) was used for data entry and statistical analysis.

## Results

### Participant Characteristics

Of the 51 initial participants, 47 were included in the data analysis as they filled out both questionnaires (T0 and T1). This group consisted of 25 men and 22 women with a mean (SD) age of 74 (6.2) years. All participants were of Dutch nationality and had a relatively high level of education: 18 out of 47 (38%) had a university degree or a higher professional education qualification and 19 participants (40%) had a secondary vocational education qualification. The majority of the participants (32/47, 68%) lived with a partner, either married or unmarried. The average grade that participants gave for their own health was 7.6 (range 0-10, higher scores indicating better health). Only 14 participants (30%) indicated that they felt slightly hindered in their daily activities due to a long-term illness or disability.

### Use of Technology and Internet

All 47 participants were asked to indicate the extent to which they adopted certain devices. A smartphone was used “regularly” by 11 participants (23%) and “daily” by 18 participants (38%). An iPad or tablet was used “regularly” or “daily” by 26 participants (55%), and a laptop was used “daily” by 26 participants (55%). All participants indicated that they used the internet. Furthermore, 31 out of the 47 participants (66%) considered its use to be “(very) easy,” 12 (25%) participants found the easiness of internet use as neutral, and only 4 participants (9%) found the use of internet “difficult.” In addition, 30 participants (65%) considered the internet to be “(very) important for keeping in touch with others.”

### Informal Care

Out of the 47 participants, 13 (28%) indicated that they helped someone living in their immediate vicinity. A considerably larger proportion (31/47) was prepared to offer help and the majority (43/47) was willing to accept help. However, 39 (83%) participants indicated that they did not need any help from people living in their immediate vicinity (see Table 2).

**Table 2:** Participants’ views on informal care (n=47).

Variables			Value
**Gave help to someone, n (%)**			
	Yes	13 (28)	
	No	33 (70)	
	Missing (no answer)	1 (2)	
**Prepared to offer help, n (%)**			
	Yes	31 (66)	
	No	6 (13)	
	Missing (no answer)	10 (21)	
**Willing to accept help, n (%)**			
	Yes	43 (92)	
	No	3 (6)	
	Missing (no answer)	1 (2)	
**Need help, n (%)**			
	Yes	7 (15)	
	No	39 (83)	
	Missing (no answer)	1 (2)	

### Community Involvement and Related Needs

The extent to which participants felt connected to people in their immediate vicinity was high; 20 out of 47 participants (43%) felt connected with most people and 13 participants (28%) with almost everyone. Only 5 participants (11%) indicated a need for more contact with people from their immediate vicinity. The vast majority, that is, 33 out of 47 participants (70%) specified that they felt no need for more contacts as they were satisfied with the number of contacts they already had. These results show that, in general, the participants already had a relatively large social network and felt no need to expand it further.

### Use of the Grubbenvorst-Online Community Care Platform

The mean (SD) score (range 0-10, higher scores indicating greater valuation) that the participants gave to the online community care platform as a whole was 7.3 (1.0) at T0 and 7.1 (1.3) at T1. Table 3 gives an overview of the use of the Grubbenvorst-Online platform as a whole (see row and per functionality at baseline, T0, and at follow-up, T1). The functionalities “Grubbenvorst-Online,” “Columns and interviews,” “Photo and Video,” and “Messages” were the most frequently used at both T0 and T1. However, only a minority of the participants indicated that they used the aforementioned functionalities on a regular or daily basis. The functionalities “Dialect Dictionary,” “Social Services,” “Local entrepreneurs,” “Local associations,” “Health care services,” and “Contacts” were the least used.

In general, a minor decrease was reported in the use at the level of specific functionalities: at T1, the “regular” use of functionalities decreased or remained stable while the “daily” use of functionalities did not occur (with the exception of 2 participants who consulted the Grubbenvorst-Online feature).

**Table 3:** Self-reported use of the online community care platform Grubbenvorst-Online as a whole and per functionality (n=47).

Online community care platform/functionality			Regular use		Daily use
**Grubbenvorst-Online (platform), n (%)**					
	T0^a^	17 (36)		4 (9)	
	T1^b^	11 (23)		3 (6)	
**Grubbenvorst-Online (functionality), n (%)**					
	T0	14 (30)		1 (2)	
	T1	12 (26)		2 (4)	
**Columns and interviews, n (%)**					
	T0	12 (26)		0 (0)	
	T1	7 (15)		0 (0)	
**Calendar Grubbenvorst, n (%)**					
	T0	8 (17)		0 (0)	
	T1	8 (17)		0 (0)	
**Photo and video, n (%)**					
	T0	11 (23)		0 (0)	
	T1	11 (23)		0 (0)	
**Dialect dictionary, n (%)**					
	T0	3 (6)		0 (0)	
	T1	2 (4)		0 (0)	
**Social services, n (%)**					
	T0	2 (4)		0 (0)	
	T1	2 (4)		0 (0)	
**Local entrepreneurs, n (%)**					
	T0	2 (4)		0 (0)	
	T1	0 (0)		0 (0)	
**Local associations, n (%)**					
	T0	4 (9)		0 (0)	
	T1	4 (9)		0 (0)	
**Health care services, n (%)**					
	T0	4 (9)		0 (0)	
	T1	2 (4)		0 (0)	
**Contacts, n (%)**					
	T0	6 (13)		0 (0)	
	T1	2 (4)		0 (0)	
**Messages, n (%)**					
	T0	15 (33)		2 (4)	
	T1	12 (26)		0 (0)	
**Help each other, n (%)**					
	T0	6 (13)		0 (0)	
	T1	6 (13)		0 (0)	

^a^T0: baseline questionnaire.

^b^T1: questionnaire after 4 months of the study.

### Impact of the Grubbenvorst-Online Platform: Expectations and Experiences

Table 4 shows the number of participants who (fully) agreed at T0 with propositions about various potential effects of their use of Grubbenvorst-Online; in other words, these correspond to the participants’ expectations of the platform. Table 4 also shows the number of participants who (fully) agreed with the same propositions about Grubbenvorst-Online at T1: this is the participants’ perception of the impact of the platform after having used it. In general, the participants’ expectations of Grubbenvorst-Online were not fully met. At T1, the participants’ overall perceived impact of Grubbenvorst-Online was significantly lower than they had expected with respect to “information provision about Grubbenvorst” (*P*=.003), “seeking help from fellow villagers” (*P*<.001), “giving help to fellow villagers” (*P*<.001), and “consulting care or welfare services” (*P*<.001). Their expectations of Grubbenvorst-Online and its perceived impact differed least regarding “participating in local activities,” “feeling connected to Grubbenvorst,” and “expansion of social network.” Overall, participants perceived the highest impact of Grubbenvorst-Online regarding “information provision about Grubbenvorst,” “feeling connected to Grubbenvorst,” and “participating in local activities.”

**Table 4:** Expectations of Grubbenvorst-Online (T0) and the perceived impact of Grubbenvorst-Online (T1) regarding various indicators.

Variables		Value (n=47)	*P* value
**Information provision about Grubbenvorst, n (%^a^)**			.003^b^
	Expectation	36 (77)	
	Perceived impact	24 (51)	
**Participating in local activities, n (%)**			.23
	Expectation	14 (30)	
	Perceived impact	9 (19)	
**Feeling connected to Grubbenvorst, n (%)**			.18
	Expectation	19 (40)	
	Perceived impact	14 (30)	
**Expansion of social network, n (%)**			.11
	Expectation	13 (28)	
	Perceived impact	6 (13)	
**Seeking help from fellow villagers, n (%)**			<.001^b^
	Expectation	17 (36)	
	Perceived impact	2 (4)	
**Giving help to fellow villagers, n (%)**			<.001^b^
	Expectation	17 (36)	
	Perceived impact	3 (6)	
**Consulting care or welfare services, n (%)**			<.001^b^
	Expectation	22 (47)	
	Perceived impact	1 (2)	

^a^Strongly agree with proposition.

^b^This value was significant at *P*<.05 in the McNemar test (two-sided).

## Discussion

### Major Findings

We explored the self-reported use, expectations, and perceived impact among older adults of a Dutch online community care platform. The study involved 47 healthy and predominantly “internet-skilled” older users (average age, 74 years). The vast majority of these users indicated that they were willing to help people living in their immediate vicinity; however, they did not necessarily feel a “help need” themselves. Furthermore, only a small proportion indicated a need for more social contact. The online community care platform was graded by the participants with an overall “more than sufficient” grade of 7.2. In general, the use of the online community care platform decreased during the 4-month follow-up period. The functionalities “Grubbenvorst-Online,” “Messages,” “Photo and Video,” and “Columns and interviews” were the most frequently used. At follow-up, participants’ perceived impact of Grubbenvorst- Online was significantly lower than their initial expectations of the impact the platform would provide regarding “information provision about Grubbenvorst,” “seeking help from fellow villagers,” “giving help to fellow villagers,” and “consulting care or welfare services.”

When the participants’ perceived impact of Grubbenvorst-Online is viewed in isolation (ie, without comparing it to their expectations), it can be ascertained that participants perceived the highest impact with respect to “information provision about Grubbenvorst,” “feeling connected to Grubbenvorst,” and “participating in local activities.” This indicates that an online community care platform can play a role in stimulating positive feelings toward communities and encouraging its members to undertake activities locally.

It is noteworthy that participants perceived little-to-no impact regarding (mutual) informal caregiving (ie, asking for or giving help to fellow villagers), although there was a high degree of willingness on the part of these participants to care for or accept help from people living in their vicinity. However, the participating older adults did indicate that they felt no need to expand their networks and did not express any great need for care from people living in their vicinity. Therefore, perhaps this limited perceived impact regarding (mutual) informal caregiving is related to the limited need for support or network expansion. This finding is remarkable as these web-based communities or platforms aim to support (and stimulate) older adults to exchange various forms of social support as it is increasingly expected that they must rely on their social network. This finding implies that online community care platforms are not suited for every older adult as such platforms have goals that not every older adult will share; not all older adults need web-based support or require help to find offline support or to request the assistance of others.

Based on our findings, we can conclude that older adults who are still healthy, self-reliant, and internet-skilled seem to be able to find their own means of solving problems and arranging informal care. This is not new because if governments or other organizations that bear responsibility for the welfare of citizens fail to provide people with a sense of security (eg, when state provision is reduced and as a result, older adults are increasingly expected to take care of themselves instead of relying on health care services), people will feel compelled to organize this security themselves. In this scenario, they will look for and find solidarity in small-scale physical groups [19]. It may therefore not be desirable at all to “formally” stimulate or organize local participation or mutual informal care; perhaps, platforms should merely try to provide another means by which citizens themselves can organize local participation and informal care.

### Strengths and Limitations of This Study

The strength of this study is that it contributes to knowledge about the (perceived) impact of a web-based community and about its actual use after the implementation. However, we did not intend to demonstrate actual causality between the functionalities of the web-based platform and their intended outcomes (eg, an increase in local participation or feelings of local connectedness) but instead aimed to illustrate how older users perceive the use and impact of an online community care platform. Furthermore, the results should be interpreted with some caution considering the modest sample size and the possible “biased sample:” participants were mainly healthy, highly educated, and internet-skilled. Since the primary target groups of the platform were older adults and vulnerable inhabitants of Grubbenvorst, we did not include the target group of the platform in its entirety, as vulnerable people were not included in the sample. Moreover, as the purpose of this study was to understand the expectations and perceived impact of a relatively small group of users regarding a web-based platform, it might have been of added value to additionally adopt qualitative methods to answer our research questions.

Additionally, it is possible that the “first-come-first-served principle” as adopted in the recruitment process resulted in a biased sample in which users who used Grubbenvorst-Online more than others registered to participate in the study. Perhaps, because of this, the depiction we now have of the use of Grubbenvorst-Online is more positive than actually is the case. Furthermore, the strength of our study would have been improved if we had asked participants the length of time they had been using Grubbenvorst-Online since expectations of new users would possibly be different from the expectations of a long-term user. Finally, in the questionnaire, we did not ask the types of help participants were willing to offer or accept. Thus, we cannot be certain whether older adults do not need support or informal care or whether they have simply already arranged it themselves without the help of a web-based platform or other tool.

### Implications for Practice and Research

Previous research demonstrates that various organizations and governments implement online community care platforms with the aim of supporting (vulnerable) citizens [3,5-9]. Based on our findings, we must ask ourselves whether such platforms provide enough “added value” in their current form. Perhaps another direction is desirable, one in which online community care platforms primarily support existing offline initiatives to stimulate local participation, informal caregiving, and feelings of connectedness. An online community care platform would thus never be considered an end in itself, but as a means to achieve certain goals. We believe that online community care platforms can add value when they encompass the following characteristics: (1) the platform’s primary objective is to provide access to offline services in a neighborhood, (2) the platform mainly has a facilitating and intermediary role and, for example, connects “help requests” of older adults or (vulnerable) citizens with available local resources (eg, other citizens, volunteers, and professionals from health care organizations), and (3) human representatives of the platform are physically present in the neighborhood during set times, and offline communication (eg, by telephone or face-to-face interactions) is the primary form by which older adults may voice their help requests. In this model, the idea of a “one-stop shop” becomes the guiding principle underlying an online community care platform, that is, all local help requests and local resources from a neighborhood come together in one place.

Additionally, previous research indicates that many (eHealth) apps (such as certain functionalities of the online community care platform) lack a clear theoretical basis and do not provide any evidence concerning their effectiveness and usability [20]. In the further development of online community care platforms, it is advisable to pay more attention to the theoretical “rationale” of the platform and its functionalities. Another important recommendation regarding the future development and implementation of online community care platforms is to involve potential end users. Previous research stresses the importance of including users to constitute web-based communities that will be functional, usable, and accessible [11,21,22]. Finally, regarding the future development of online community care platforms, it is advisable that developers continually rethink the platforms’ features, make adjustments, and evaluate whether the functionalities are actually effective.

Further research is required to establish if online community care platforms have an impact on older adults’ local participation, informal caregiving, and feelings of connectedness, and if so, the ways in which this impact is felt. Research should focus on the impact of different online community care platforms and on their impact on the group of older adults in all its variety: youngest-old, middle-old, and oldest-old; frail and healthy; highly and poorly educated; and internet-skilled and unskilled. Finally, since platforms often focus on all inhabitants of a neighborhood to create an interdependent support system where old and young people are facilitated to help each other, further work should also focus on researching the impact of these platforms on user groups other than older adults.

Finally, it would be interesting to study the impact of the current coronavirus pandemic on the adoption and use of web-based platforms. Perhaps the pandemic may lead to an increased use of these platforms, as it has led to a striking adaptation of various eHealth services in community care (which used to have a history of strenuous and slow adoption and implementation).
